# The study of differences by region and type of gambling on the degree of gambling addiction in Japan

**DOI:** 10.1038/s41598-021-92137-8

**Published:** 2021-06-23

**Authors:** Shingo Hayano, Ran Dong, Yoshie Miyata, Sakutaro Kasuga

**Affiliations:** 1 Tsuru University, Faculty of Letters, Yamanashi, 402-8555 Japan; 2 Tokyo University of Technology, School of Computer Science, Tokyo, 192-0982 Japan; 3 Higashiyamato Municipal 10th Elementary School, Tokyo, 207-0023 Japan

**Keywords:** Quality of life, Health policy, Addiction

## Abstract

We conducted an online national survey using the South Oaks Gambling Screen (SOGS) on 42,880 people in Japan to identify gambling addiction prevalence differences with regard to gambling type and region. This study included 14,780 individuals (valid responses) who engaged in gambling activities in the past year. There was a large difference in mean SOGS score by gambling type: public gambling and casinos score ≈ 4.0, whereas lotteries (including numbers, etc.) ≈ 1.43. SOGS scores were much higher for those who participated in more than one gambling type than for those who participated in only one type. Regional differences in SOGS scores were also confirmed, with more populous prefectures having lower and less populous having higher SOGS scores. Integrating SOGS scores with existing data concerning hobbies and recreational behaviors across regions indicated that regions characterized by lower SOGS scores were also characterized by greater activity for hobbies and recreational behaviors.

## Introduction

Japan passed the Integrated Resort (IR) Bill in 2018, which attracted considerable attention due to its attempt to institutionalize casinos, which were currently illegal. Gambling addiction has become a hot topic in mass media since the IR Bill started to be considered. The Ministry of Health, Labor, and Welfare (MHLW) and Kurihama Medical and Addiction Center started a large-scale survey on gambling addiction in response to the IR Bill, probing how adding a new gambling type (casinos) in Japan would affect gambling addiction. The next concern was where to build casinos, with candidates including Tokyo-Odaiba, Kanagawa-Yokohama, Aichi-Tokoname, and other areas.

However, there has been little research on the relationship between regional characteristics and gambling addiction. Japan shows large regional differences in lifestyle and language^1^. The main purpose of the present study is to clarify gambling addiction differences with respect to gambling type (including casinos) and region.

Many previous studies have considered gambling addiction prevalence around the world. LaPlante et al.^2^ investigated gambling addiction in the UK from September 2006 to March 2007 through a questionnaire. Welte et al.^3^ investigated addiction status in the United States using SOGS, and Binde et al.^4^ investigated addiction status for Sweden. Calado and Griffiths^5^ summarized 69 studies from academic databases, the internet, and government websites and showed wide variations in previous year’s problem gambling rates worldwide (0.12–5.8%) compared with European countries (0.12–3.4%). However, they also noted that it was difficult to directly compare studies due to methodological procedure, scale, cutoff, and time frame differences.

As discussed above, large-scale gambling addiction research is now being conducted in Japan in connection with the IR Bill. The MHLW Scientific Research Grant 2008 Survey^6^ (MHLW Survey 2008) surveyed 7500 people (4123 valid responses), and the MHLW Scientific Research Grant 2013 Survey^7^ (MHLW Survey 2013) surveyed 7052 people (4153 valid responses). However, these surveys examined prevalence in the entire lifetime instead of prevalence in the past year. Large-scale surveys of people who gambled in the past year include the Kurihama Medical and Addiction Center 2016 Survey (Kurihama Survey 2016)^a^ (a–e: please see supplementary information), which surveyed 2200 people (993 valid responses) in 11 cities; and the Kurihama Medical and Addiction Center 2017 Survey (Kurihama Survey 2017)^b^, which surveyed 10,000 people (4685 valid responses) nationwide. All these previous studies in Japan used the South Oaks Gambling Screen (SOGS) for gambling screens.

There was also a survey limited to pachinko and pachislot (pachinko)^c^, the 2017 Survey of the Nikkoso Research Foundation for Safe Society (Nikkoso Survey 2017)^8^. Nikkoso Survey 2017 included 9000 people (5060 valid responses), but although the survey methodology was sound, it cannot be easily compared with other large-scale surveys because it used a unique method to measure gambling addiction.

The prevalence of people who gambled in the past year was 5 of 993 (0.6%) for Kurihama Survey 2016, 32 of 4685 (0.8%) for Kurihama Survey 2017, and 21 of 5060 (0.4%) for Nikkoso Survey 2017. The surveys revealed gambling prevalence in Japan, but sample sizes for problem gamblers were very small.

Previous surveys did not report gambling type or regional differences. Furthermore, sample sizes for problem gamblers (including gambling addicts) were too small to analyze regional differences with regard to gambling addiction. To the best of our knowledge, no previous studies have considered regional differences in gambling addiction across a single country.

Random sampling is mathematically guaranteed to estimate overall population response within a margin of error, but only when the (valid) response rate is close to 100%. Response rates for Kurihama Survey 2016 and Kurihama Survey 2017 = 45.1% and 46.9%, respectively, which are not statistically random and should probably be considered as biased^9,10^.

Response rates for MHLW Surveys 2008 and 2013 = 55.0% and 58.9%, respectively. Their reliability has been questioned due to high lifetime prevalence cases, estimated at 5.56 million (9.6% for men and 1.6% for women) and 5.36 million (4.8%), respectively^11^. This is because Kurihama Surveys 2016 and 2017 estimated “lifetime” prevalence of 2.8 million (2.7%) and 3.2 million (3.6%), respectively, significantly lower than MHLW Surveys 2008 and 2013. However, Kurihama Surveys 2016 and 2017 also had low response rates (45.1% and 46.9%, respectively). Although estimated population prevalence differed markedly, response rates also differed. Thus, MHLW Surveys 2008 and 2013 may have found higher prevalence due to the response rate.

The above surveys used the basic resident register to select respondents such that researchers could easily obtain personal information, including address, age, gender, etc. However, sensitive items, e.g., smoking and salary details, have higher concealed response rates when researchers obtain participant personal information directly. Hence, there may be a tendency to avoid answering^12^. Gambling addiction is considered a very sensitive item, and concealment rates are likely to be high.

## Method

Online multi-person surveys are superior to random sampling surveys to reveal regional differences regarding the degree of gambling addiction. Although the number of respondents is limited to online users, which tends to be biased toward those who are interested in the survey subject^13^, the survey’s anonymity makes it easier for respondents to express their true feelings^14^. Therefore, online surveys are considered effective when dealing with sensitive items, such as gambling addiction.

We conducted an online nationwide survey of people in their 20s to 80s using the SOGS^15^ as the gambling addiction metric (Hayano Survey 2020). Most large-scale studies conducted in Japan used SOGS, and we also used SOGS to compare our results with previous studies.

Hayano Survey 2020 was conducted from August 12 to 15, 2020, with the questionnaire distributed to 492,963 monitors in their 20s to 80s (Table 1). We completed data collection when the number of respondents who had gambled^5^ in the past year reached 15,000. Total sample size = 42,880 people, including 14,780 (valid samples) individuals who had gambled in the past year. The purpose was to analyze active gamblers who had gambled within the past year, excluding those who had gambled but not in the past year or had never gambled. Since we did not use random sampling, there was bias toward respondents with no gambling experience in each prefecture. This ensured that gambling experience conditions affecting SOGS scores were constant. The survey included not only public games, pachinko, and casinos but also lotteries and securities transactions, which have not been reported in Japan previously. Survey items identified gambling frequency, expected winnings, and reasons for gambling, but only SOGS items were used in this study.

**Table 1:** Relevant previous surveys in Japan.

	MHLW	MHLW	Kurihama	Kurihama	Nikkoso	Hayano
Survey year	2008	2013	2016	2017	2017	2020
Survey area	Nationwide	Nationwide	11 cities	Nationwide	Nationwide	Nationwide
Age range	20–	Undisclosed	Undisclosed	20–74	18–79	20–89
Survey method	Random sampling	Random sampling	Random sampling	Random sampling	Random sampling	Online
Sample size	7500	7052	2200	10,000	9000	492,963
Valid responses	4123	4153	993	4685	5060	42,880
Participants in the past year	Not surveyed	Not surveyed	Undisclosed	Undisclosed	582	14,780
Prevalent (disability)	M9.6% F1.6%^a^	4.8%^a^	26 (2.7%)	158 (3.6%)	Not surveyed	Not surveyed
Prevalent (past 1 year)	Not surveyed	Not surveyed	5	32	21	1826

^a^Only percentages are reported.

Hayano Survey 2020 was based on the modified Japanese version of SOGS^16^, and a web version was used with expressions changed to a gentler form of Japanese to make it easier for respondents to understand without changing the original meaning (Table 2). To eliminate unreliable responses, we included a dummy question (Question 12), which ascertained whether the respondents had ever been in debt due to gambling^d^. We excluded 220 respondents from further analysis who answered Question 12 negatively, but whose answers to Questions 10 and 13 indicated that they did have debt experience. We followed Lesieur and Blume^15^ guideline that respondents with SOGS score ≥ 5 were considered to have a gambling problem, treating SOGS score ≥ 5 as suggesting a gambling problem; but since we only used SOGS, we considered this to indicate a possible problem rather than definitive.

**Table 2:**
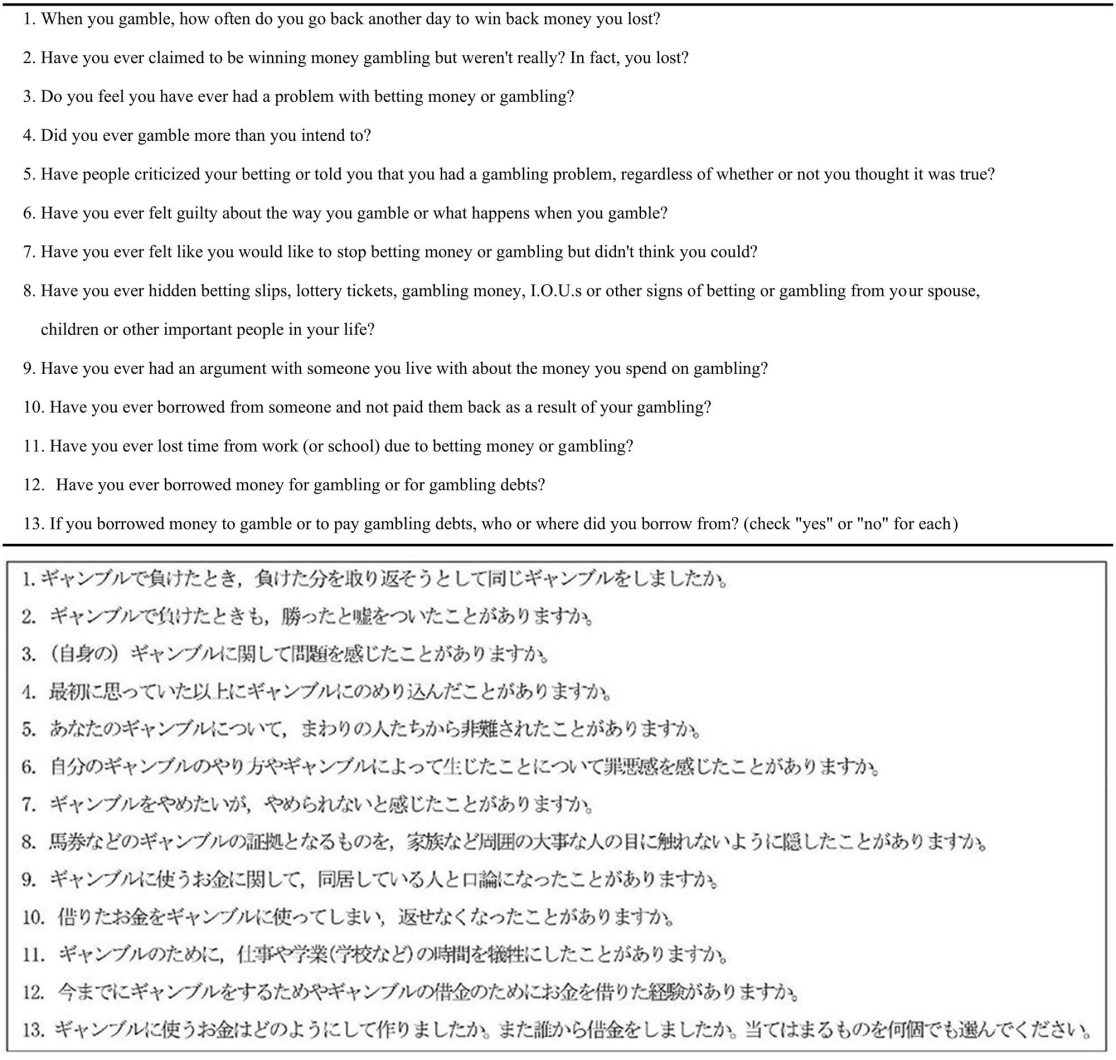
English and modified Japanese web version of SOGS.

### Ethics

This work was performed by ASMARQ who has acquired ISO20252 (Market, opinion, and social research, including insights and data analytics-vocabulary and service requirements). All methods were carried out in accordance with the relevant guidelines and regulations of the Japan Marketing Research Association (JMRA). All experimental protocols were approved by the Ethics Department of ASMARQ (Reference number: 38489). Informed consent was obtained from all subjects before starting the internet survey.

## Results

### SOGS score with respect to gambling type

**Figure 1:**
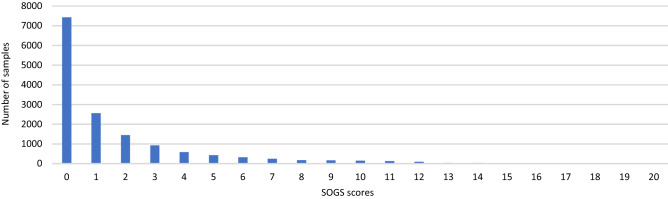
SOGS score distribution (N = 14,780)^17^.

Figure 1 shows that SOGS scores for the 14,780 valid respondents formed an inverted-J distribution^17^, and Table 3 confirms large differences in SOGS scores with respect to gambling type.

**Table 3:** SOGS scores for each gambling type.

	Overall	Horse racing	Bicycle racing	Boat racing	Auto racing	Lottery trading	Pachinko	Casino	Securities
Number of samples (n)	14,780	4538	1087	1119	592	10,088	4104	297	2134
SOGS score	1.68	2.36	3.76	3.61	4.35	1.43	3.13	3.88	2.25
Difference^a^	–	0.68	2.08	1.93	2.68	− 0.25	1.45	2.20	0.57
Standard deviation (SD)	2.71	3.24	4.15	4.00	4.48	2.55	3.46	4.36	3.24
Statistics	–	13.37	22.11	20.78	21.30	6.48	27.39	12.55	8.18
P value	< 0.001	< 0.001	< 0.001	< 0.001	< 0.001	< 0.001	< 0.001	< 0.001	< 0.001

^a^Difference: the difference between overall mean SOGS score and mean SOGS score for each gambling type. Standard deviation, statistic, and p-value are from the difference test.

Table 3 shows sample sizes for each gambling type, where lottery includes numbers and soccer lottery, pachinko includes pachislot, and securities margin trading includes investment in the futures market. The total number of respondents was 24,419 because multiple answers were allowed. 1826 respondents had SOGS score ≥ 5, indicating a probable gambling problem. Overall mean SOGS score = 1.68, 1.86 for males (n = 10,839) and 1.18 for females (n = 3941). The Nikkoso Survey, which used random sampling, showed 3.6% of gambling participants in the past year, whereas Hayano Survey 2020 showed 12.35%. This is most likely because many respondents are interested in the survey subject^13^. For example, pachinko participation rate ≈ 9.84% in the current study, whereas Japan overall has ≈ 6.42%^18^. Some previous studies have shown that internet users have higher gambling participation rates^19^. Alternately, it could be that the number of concealed responses^12^ decreased due to using the online survey.

The average SOGS score order was auto racing (4.35), then casinos (3.88), bicycle racing (3.76), boat racing (3.61), and pachinko (3.13). Auto racing and lottery differed by 2.92 points, and auto and horse racing differed by 1.99 points.

Respondents with SOGS ≥ 5 or more points were highest for auto racing (37.67%), then casinos (32.66%), bicycle racing (32.57%), boat racing (31.67%), and pachinko (26.31%). Horse racing (18.60%) was as low as securities margin trading (17.15%), and lottery (10.06%) was lower than securities margin trading. There was 27.61% difference between auto racing and lottery. There are 97 public gambling venues in Japan, including 25 horse racing tracks (10 for central and 15 for local races), 43 bicycle racing tracks, 24 boat racing courses, and five auto racing tracks (venue counts from the relevant gambling websites). SOGS score was highest for auto racing, which has the fewest venues.

Table 4 shows the number of gambling participants also differed by gambling type. Respondents who participated in only one gambling type were auto racing (6.42%), boat racing (7.69%), bicycle racing (9.66%), and casinos (10.77%), and comprised approximately 90% of those participants who also participated in other types of gambling. Lottery (59.89%) had few participants in other gambling types, and approximately 67% of pachinko players participated in other forms of gambling. Many previous studies^6^ proposed pachinko as the main cause for gambling addiction in Japan, and this situation did not occur in this survey.

**Table 4:** Number of participants and SOGS scores by gambling type (n = 14,780).

	Horse racing	Bicycle racing	Boat racing	Auto racing	Lottery trading	Pachinko	Casino	Securities
Average number of participants	2.60	4.39	4.32	5.36	1.78	2.53	5.22	2.50
Correlation coefficient^a^	0.31	0.19	0.20	0.08	0.41	0.18	0.19	0.35
Participation in type 1 (%)	27.28	9.66	7.69	6.42	59.89	33.09	10.77	43.21
SOGS (participation in 1 type)	1.50	2.33	2.66	4.66	0.70	2.77	2.25	1.46
SOGS (2 or more types)	2.68	3.91	3.69	4.33	2.52	3.31	4.08	2.84
Difference^b^	1.19	1.58	1.02	− 0.33	1.82	0.54	1.83	1.38
Statistics	12.23	5.27	3.13	0.67	30.79	5.57	3.35	10.85
P value^c^	< 0.001	< 0.001	0.035	0.993	< 0.001	< 0.001	0.018	< 0.001

^a^Correlation coefficient between average number of participants and SOGS scores.

^b^Test for differences between single type and two or more types.

^c^Boat racing and casinos significant at p < 0.05, auto racing not significant.

The correlation coefficient between the number of gambling types participated in and SOGS scores = 0.30. In horse racing, bicycle racing, boat racing, lotteries, casinos, and securities trading, tended to increase SOGS scores by more than 1 point, when participating in other types of gambling. Participants in auto racing had higher SOGS scores regardless of the number of gambling types they participated in.

### Regional differences in SOGS scores

**Figure 2:**
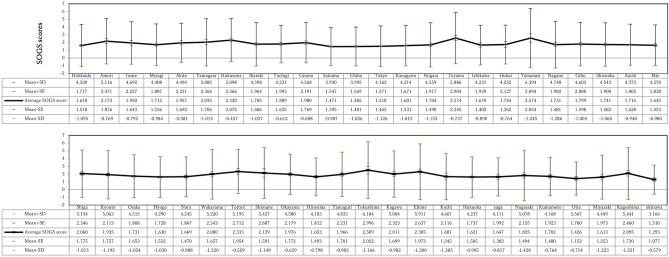
Mean values for each prefecture level.

Figure 2 shows average SOGS scores, mean ± SD (standard deviation), and mean ± SE (standard error) by prefecture. In descending order: Toyama, Yamanashi, Tokushima, Tottori, Fukushima, Ehime, Shimane, and Kagoshima had mean SOGS scores = 2.57, 2.57, 2.51, 2.33, 2.32, 2.31, 2.14, and 2.10, respectively.

Scores were high in Yamanashi, Tottori, Shimane, and Kagoshima prefectures, which do not currently have public gambling. On the other hand, Saitama and Fukuoka prefectures, which both have public gambling, exhibit low scores 1.47 and 1.62, respectively, and Tokyo was also low (1.52). Other prefectures with large cities (Miyagi, Kanagawa, Aichi, Osaka, and Hyogo) exhibit SOGS scores = 1.71, 1.60, 1.72, 1.72, and 1.63, which are all close to the overall average. Thus, there were regional differences in SOGS scores, and values tended to be higher in regions with smaller populations. Prefectures with smaller populations tended to have larger margins of error due to smaller sample sizes.

Yamanashi had the largest error and the smallest sample size. Table 5 shows multiple comparisons between Yamanashi (n = 54); Toyama (n = 101), which had small samples and high SOGS scores; and the three capital prefectures (Tokyo, Chiba, and Saitama), confirming significant differences. The correlation coefficient = − 0.443 between SOGS scores by prefecture and population data by prefecture published by the Statistics Bureau of the Ministry of Internal Affairs and Communications^20^, indicating a significant correlation. Overall average SOGS score = 2.09 for prefectures with populations less than one million. Comparison with Tokyo’s average SOGS (1.52) was significant at p < 0.001 (Student’s-t statistic = 4.05, degrees of freedom = 2946). All comparisons between prefectures with less than one million population and Miyagi, Aichi, Osaka, and Fukuoka (prefectures with large cities) were also statistically significant (average SOGS were lower in prefectures with large cities).

**Table 5:** Multiple comparison tests for Yamanashi, Toyama, and Tokyo metropolitan area.

Prefecture1	Prefecture2	Meanl	Mean2	Difference	Statistics	P-value
Yamanashi (n = 54)	Saitama (n = 1046)	2.5741	1.4713	1.1028	4.2143	0.0195*
Yamanashi	Chiba (n = 889)	2.5741	1.4848	1.0893	4.2484	0.0170*
Yamanashi	Tokyo (n = 2515)	2.5741	1.5181	1.0560	4.0972	0.0306*
Toyama (n = 101)	Saitama (n = 1046)	2.5743	1.4713	1.1029	6.9162	0.0000**
Toyama	Chiba (n = 889)	2.5743	1.4848	1.0894	6.8976	0.0000**
Toyama	Tokyo (n = 2515)	2.5743	1.5181	1.0562	6.7835	0.0000**

*P < 0.05, **P < 0.01.

**Figure 3:**
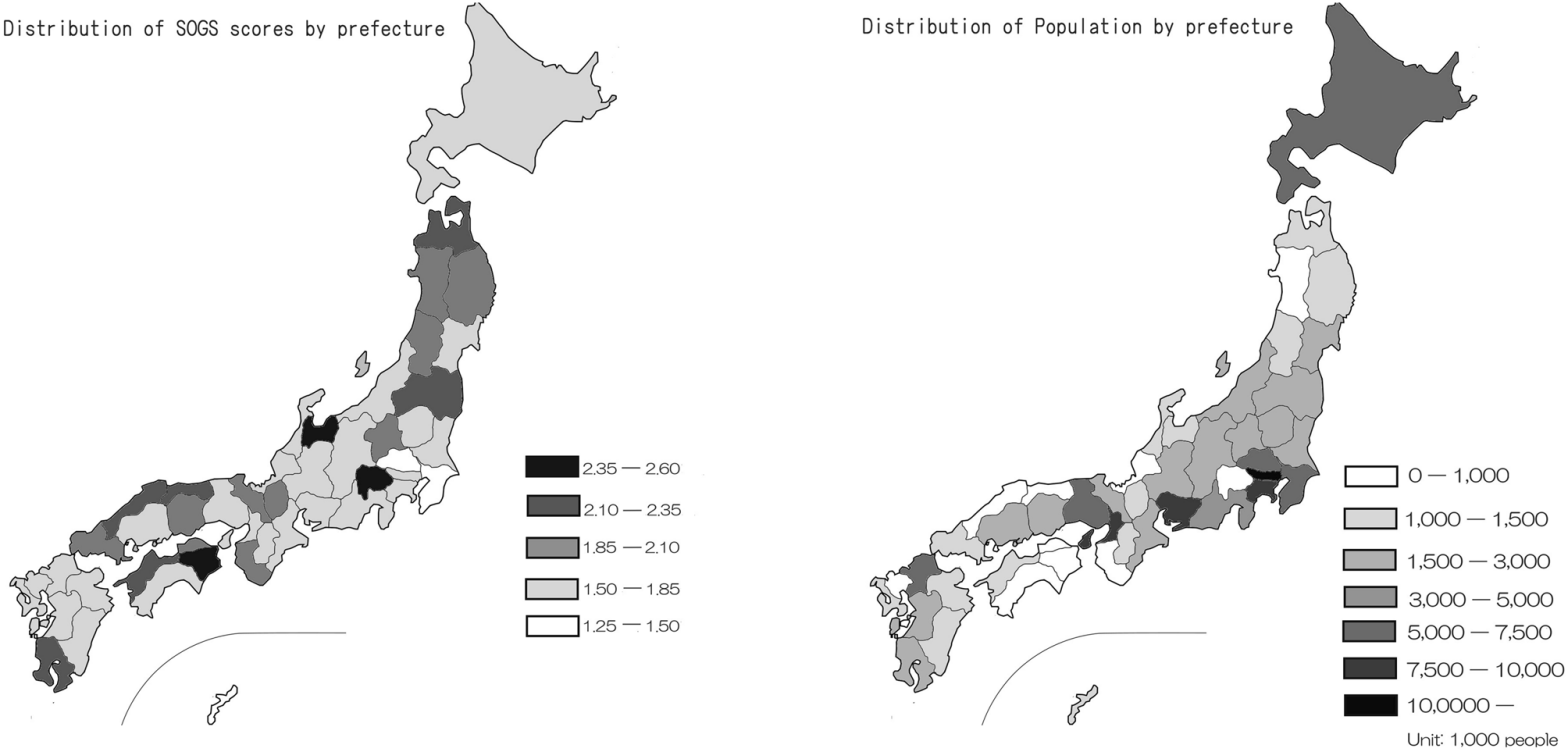
SOGS and population distributions (made using Zoner Photo Studio 18 (Ver. 0.19) https://www.zoner.com/).

Figure 3 shows SOGS distributions from Hayano Survey 2020 and population distributions by prefecture published by the Statistics Bureau of the Ministry of Internal Affairs and Communications^20^. SOGS scores were higher for areas with smaller populations.

### Factors contributing to regional differences in degree of gambling addiction

Correlation coefficient = − 0.398^d^ between the number of public gambling venues by prefecture and average SOGS by prefecture, indicating low negative correlation, i.e., increased public gambling venues tended to lower SOGS scores. This may be because more populated prefectures have more public gambling venues (correlation coefficient = 0.751 between public gambling halls and population by prefecture). This is particularly the case on a prefectural basis (macro perspective), and previous studies have reported that addiction ratio increased within a few kilometers from gambling areas (micro perspective)^21^. Correlation coefficient = − 0.434 between the number of pachinko parlors and SOGS score by prefecture (National Police Agency’s Security Division, 2019), which is a substantial correlation, i.e., prefectures with more pachinko parlors tend to have lower SOGS. This is also related to the population by prefecture rather than the number of pachinko parlors (correlation coefficient = 0.97 between population by prefecture and number of pachinko parlors). This suggests there are factors that counteract partial negatives in populated areas, such as the presence of gambling venues (including gambling machines). We assume a major factor is a recreational behavior.

The ratio of population to each pachinko machine is high in Tokyo (42.3) and Kanagawa Prefecture (40.7), which had low SOGS, and low in Toyama (26.8), Yamanashi (29.6), Tokushima (24.7), Tottori (22.6), Fukushima (23.5), and Kagoshima (19.0), which had high SOGS (National Police Agency’s Security Division, 2019). Prefectures with smaller populations have lower population–pachinko machine ratios. Correlation coefficient = − 0.327 between population ratio and SOGS, indicating a correlation. Thus, increased pachinko machines per capita tends to increase SOGS. The number of pachinko parlors is higher in more populated areas, but the situation changes when the number is calculated as a percentage of the population (15 years old or older). Kagoshima, Miyazaki, Tottori, and Shimane have approximately one pachinko parlor per 6220, 7180, 7200, and 8010 people, respectively; whereas Tokyo, Kanagawa, Chiba, and Saitama have approximately one pachinko parlor per 14.930, 15.120, 13.230, and 13.130 people, respectively. Correlation coefficient = − 0.34 between the number of pachinko parlors and SOGS as a percentage of the population. Increased number of pachinko parlors and pachinko machines relative to the population ratio in each prefecture tends to increase SOGS scores. Gambling addiction needs to be considered not simply in terms of the number of gambling venues and gambling machines, but also in terms of the number of gambling venues and gambling machines relative to the population.

Comparing previous survey reports confirms that more populated areas have lower SOGS scores. Kurihama Survey 2016, conducted in large cities such as the 23 wards of Tokyo, Yokohama, Osaka, Nagoya, and Fukuoka, reported 2.7% (approximately 2.8 million people) for a lifetime and 0.6% (approximately 600,000 people) for the past year. Kurihama Survey 2017, which surveyed the entire country, reported 3.6% (approximately 3.2 million people) and 0.8% (approximately 700,000 people), respectively. Kurihama Surveys 2016 and 2017 confirm prevalence or problem of gambling is lower in urban areas.

### Entertainment, hobby activities, and gambling addiction

We examined the relationship between gambling addiction and entertainment. The Basic Survey on Social Life^22^, a nationwide survey conducted every five years by the Statistics Bureau of the Ministry of Internal Affairs and Communications (MIC), includes recreational activity rates by prefecture and hobby or pastime type regarding entertainment and hobby behaviors. We targeted approximately 200,000 household members aged 10 years or older in approximately 88,000 randomly selected households in the designated survey areas (approximately 7300 survey areas nationwide). The survey was conducted from October 15 to October 23, 2016. The Basic Survey on Social Life reports the “rate of respondents by type of hobby or pastime by prefecture—total number of males and females (15 years and older).” The rate of people who engaged was calculated by dividing the number of people who engaged in each activity type in each prefecture in the past year by the population. The number of samples in each prefecture ranges from 2801 to 5837, indicating the percentage of prefectural residents who engaged in that recreational activity on a prefectural basis. The Basic Survey on Social Life, Hayano Survey 2020, number of gambling parlors, number of pachinko parlors, and number of pachinko machines were all reported separately, but since they were all surveyed on a prefectural basis, we calculated correlation coefficients for each prefecture as a unit (sample), i.e., 47 samples in total.

Correlation coefficient = 0.652 between recreational activity rate (percentage of people who took part in recreational activities) and prefectural population (15 years old and over). Thus, more populous prefectures tend to perform more entertainment activities. Correlation coefficient = 0.521 and 0.581 between the number of public gambling venues and recreational activity rate; and the number of pachinko parlors and recreational activity rate.

Correlation coefficient = 0.569 between population–pachinko machine ratio and recreational activity rate, hence regions with lower levels of entertainment behaviors have more pachinko machines per capita. It is likely that pachinko accounts for a higher proportion of entertainment behaviors in regions with lower populations.

Table 6 shows correlation coefficients between each prefecture’s values (rate of people who engaged) on entertainment items from the Basic Survey on Social Life and average SOGS for each prefecture in Hayano Survey 2020 (treating each prefecture as one unit)^e^. The signs are all negative, indicating that provinces, where these pastimes and hobbies are popular, have lower SOGS scores. For example, correlation coefficient = − 0.501 for karaoke, calculated from karaoke participation rate and SOGS for each prefecture, i.e., SOGS is low for prefectures where many people participate in karaoke. SOGS scores are low for prefectures with many people who participate in Western dancing, ballroom dancing, and camping. These pastimes are often carried out with others, but we can speculate that recreational activities that are combined with others may help prevent gambling addiction. In particular, hobbies that require communication with others have low SOGS scores, but solitary hobby activities such as photography and photo printing, and reading as a hobby may also help prevent gambling addiction.

**Table 6:** Correlations between entertainment/hobby activities and SOGS scores (n = 47).

Entertainment item	Correlation coefficient
Karaoke	− 0.501
Listening to music on CD, smartphone, etc.	− 0.454
Western and ballroom dancing	− 0.449
Camping	− 0.444
Watching movies outside of movie theaters (TV, DVD, computer, etc.)	− 0.415
Traditional Japanese music (including folk songs and music from ancient Japan)	− 0.414
Taking and printing photographs	− 0.386
Playing musical instruments	− 0.384
Movies at the cinema	− 0.360
Reading as a hobby	− 0.306
Cooking and baking as a hobby	− 0.345
Painting and sculpture	− 0.341
Listening to popular music or songs at music concerts	− 0.327
Watching sports (excluding TV, smart phone, computer, etc.)	− 0.325
Watching entertainments, plays and dances (excluding TV, smartphone, PC, etc.)	− 0.324
Video games, computer games (including those played at home and those for cell phones)	− 0.313
Amusement park, zoo, aquarium, etc	− 0.298

The Basic Survey on Social Life also surveyed pachinko behavior. Correlation coefficient = − 0.46 between pachinko behavior rate and population by prefecture. Therefore, prefectures with smaller populations have higher pachinko rates. Correlation coefficient = 0.18 between pachinko participation and SOGS. Since the sign is positive, participation increases the tendency toward dependence, but the difference is not significant.

## Discussion

This paper conducted an online survey using SOGS to confirm gambling addiction status differences depending on gambling types practiced in Japan and regional differences. Auto racing (37.67%), casinos (32.66%), bicycle racing (32.57%), and boat racing (31.67%) had the highest percentage SOGS ≥ 5. However, prefectural SOGS tended to be higher for smaller populations, and SOGS scores were higher in Yamanashi, Tottori, Shimane, and Kagoshima prefectures, where there were no public gambling halls.

The MIC survey conducted in 2016 confirms that pachinko participation rates are higher in less populated areas. An entertainment activity survey conducted by MIC showed that entertainment activities were more popular in more populous prefectures. Hayano Survey 2020 combined SOGS by prefecture, showing that SOGS scores were lower in prefectures where karaoke, music appreciation, and Western and ballroom dancing were popular. SOGS scores were probably higher in prefectures with smaller populations because they have fewer entertainment activities but more pachinko activities, which would also explain why SOGS scores were higher in prefectures without public gambling venues. However, no significant differences were found between pachinko behavior from the MIC survey and SOGS from Hayano Survey 2020. Therefore, pachinko is not as strongly associated with gambling addiction as auto racing and bicycle racing. Gambling addiction in sparsely populated prefectures was explained in terms of pachinko-related situations, since pachinko activities were the only ones surveyed in this study, but activities such as lotteries and online gambling, which have the highest participation rates, could well be a factor. Cause-and-effect relationships between these factors would be worthwhile issues to explore in the future.

Public gambling has become more accessible than pachinko games because online gambling is available, allowing people to participate from home. Pachinko parlors were required to close during the recent Covid-19 crisis, and hence the number of online horse racing gamblers increased fivefold from the previous year while the amount of money spent per gambler also increased^23^. Since public gambling is more strongly linked to gambling addiction than pachinko, increased online gamblers pose a great danger. Casinos, which are being considered in the IR Bill, are the second most strongly linked to gambling addiction after auto racing, according to Hayano Survey 2020.

## Supplementary Information

Supplementary Information.
