# Supplementary material for: The study of differences by region and type of gambling on the degree of gambling addiction in Japan
Source: Sci Rep. 2021 Jun 23;11:13102. doi: 10.1038/s41598-021-92137-8 (PMC8222369; doi:10.1038/s41598-021-92137-8)
Supplement: Supplementary file 1 — Supplementary Information. [file 41598_2021_92137_MOESM1_ESM.docx]

**Supplementary Information**

1. The Survey results not disclosed; figures are from Mainichi Shimbun, March 31, 2017
2. The Survey results not disclosed; figures are from Mainichi Shimbun, September 29, 2017
3. The Japanese government refers to gambling addiction in medicine and psychology as “gambling and other addiction. This is because the term “gambling and other addiction” includes addiction to pachinko and pachislot, which are not legally classified as gambling but as amusement. The term “gambling” used in this paper refers not only to pachinko and pachislot but also to securities margin trading (including investment in the futures market).
4. One problem with online surveys is that there are a few samples that do not read the questions and answer all questions with the same number (e.g., all 1s). In order to eliminate such samples, we included this dummy (on the advice of the research firm that conducted this Survey).
5. Since the number of prefectures is 47(n=47), the correlation coefficient is 0.288 with p<.05 and 0.372 with p<.01
